# Relationships between oxygen changes in the brain and periphery following physiological activation and the actions of heroin and cocaine

**DOI:** 10.1038/s41598-021-85798-y

**Published:** 2021-03-18

**Authors:** Shruthi A. Thomas, Carlos M. Curay, Eugene A. Kiyatkin

**Affiliations:** Behavioral Neuroscience Branch, National Institute on Drug Abuse – Intramural Research Program, National Institutes of Health, DHHS, Baltimore, MD 21224 USA

**Keywords:** Neuroscience, Physiology

## Abstract

Using two-sensor electrochemical recordings in freely moving rats, we examined the relationship between physiological and drug-induced oxygen fluctuations in the brain and periphery. Animals chronically implanted with oxygen sensors in the nucleus accumbens (NAc) and subcutaneous (SC) space were subjected to several mildly arousing stimuli (sound, tail-pinch and social interaction) and intravenous injections of cocaine and heroin. Arousing stimuli induced rapid increases in NAc oxygen levels followed by and correlated with oxygen decreases in the SC space. Therefore, cerebral vasodilation that increases cerebral blood flow and oxygen entry into brain tissue results from both direct neuronal activation and peripheral vasoconstriction, which redistributes arterial blood from periphery to the brain. The latter factor could also explain a similar pattern of oxygen responses found in the *substantia nigra reticulata,* suggesting hyperoxia as a global phenomenon with minor structural differences during early time intervals following the stimulus onset. While arousing stimuli and cocaine induced similar oxygen responses in the brain and SC space, heroin induced a biphasic down-up brain oxygen fluctuation associated with a monophasic oxygen decrease in the SC space. Oxygen decreases occurred more rapidly and stronger in the SC space, reflecting a drop in blood oxygen levels due to respiratory depression.

## Introduction

High metabolic activity of brain cells requires continuous and adequate delivery of oxygen, which arrives into brain tissue via arterial blood. Blood oxygen levels depend on breathing and are tightly regulated, remaining relatively stable throughout the entire continuum of activity states. Although gradient-dependent entry from arterial blood is the primary factor that determines oxygen levels in brain issue, this entry can be enhanced during functional neural activation via changes in cerebral vessel tone and increases in local cerebral blood flow (CBF). This gradient-independent mechanism, usually considered in terms of neurovascular coupling^1–3^, allows the brain to receive more oxygen in advance of its enhanced consumption during functional neural activation^4–6^. Consequently, extracellular oxygen levels in brain tissue reflect a balance between highly dynamic and opposing influences of entry from arterial blood and metabolic consumption.

Considering the mechanisms underlying cerebral vasodilation and enhanced CBF, it is usually postulated that these effects result from neuronal activation that triggers the release of a number of vasoactive substances in the vicinity of cerebral capillaries^1^. However, cerebral vasodilation followed by increased CBF may also occur due to peripheral vasoconstriction—a common, centrally mediated effect triggered by functional neural activation^7–9^. Through this mechanism of blood re-distribution, first noted by Charles Sherrington^10^, the blood inflow to the brain and subsequent oxygen entry could be rapidly and substantially increased during neural activation.

To substantiate the possible role of this mechanism, it is important to examine the relationship between oxygen changes occurring in the brain and periphery. By employing oxygen sensors coupled with high-speed amperometry, we previously demonstrated that oxygen levels in the nucleus accumbens (NAc) modestly but rapidly increase following exposure to different sensory and arousing stimuli^11,12^. While it may be reasonably assumed that these oxygen increases result from neuronal activation and subsequent cerebral vasodilation and increased CBF, it is unclear how these stimuli affect oxygen levels in peripheral tissues and whether these changes contribute to brain oxygen increases.

We addressed this issue by using two-sensor oxygen recordings in awake, freely moving rats, with one sensor implanted in the NAc, a critical structure involved in natural and drug reinforcement^13,14^, and the second sensor implanted in the subcutaneous (SC) space—a densely vascularized peripheral location. We assessed changes in oxygen levels in these two locations induced by three arousing stimuli of different nature and salience (a brief auditory stimulus, tail-pinch and social interaction). In addition to physiological stimuli, we examined the relationships between central and peripheral oxygen responses induced by two prominent drugs of abuse: cocaine and heroin. While intravenous (iv) cocaine passively injected at low self-administering doses modestly increases NAc oxygen levels^15,16^, it also induces peripheral vasoconstriction^17^ suggesting its possible contribution to brain oxygen increases. In contrast to sensory stimuli and cocaine, heroin modestly increases brain oxygen levels at low reinforcing doses but induces a dose-dependent decrease followed by rebound-like increase at higher drug doses^16,18^.

Arousing stimuli and drugs both have global and structure-specific effects on neuronal activity and brain metabolism. To assess whether NAc oxygen responses are structure specific or analogous to the brain as a whole, we examined the effects of the same physiological and pharmacological stimuli on oxygen responses in the *substantia nigra pars reticulata* (SNr)—a structure with profound differences in basal and stimulus-induced impulse activity.

## Methods

### Subjects

11 adult male Long-Evans rats (Charles River Laboratories) weighing 440 ± 40 g at the time of surgery were used in this study. Rats were individually housed in a climate-controlled animal colony maintained on a 12–12 light–dark cycle with food and water available ad libitum. All procedures were approved by the NIDA-IRP Animal Care and Use Committee and complied with the Guide for the Care and Use of Laboratory Animals (NIH, Publication 865–23) and ARRIVE guidelines. Maximal care was taken to minimize the number of experimental animals and any possible discomfort or suffering at all stages of the study.

### Surgical preparations

Surgical procedures for electrochemical assessment of oxygen have been described in detail elsewhere^11,18^. Briefly put, under general anesthesia (Equithesin, a mixture of sodium pentobarbital and chloral hydrate), rats prepared for the first experiment were chronically implanted with Pt-Ir oxygen sensors (Model 7002–02; Pinnacle Technology, Inc., Lawrence, KS, USA) in two locations. The first sensor was implanted in the NAc shell [anteroposterior + 1.2 mm, mediolateral ± 0.8 mm, and dorsoventral − 7.2–7.6 mm from the skull surface, according to coordinates from the rat brain atlas^19^, while the second sensor was implanted in the SC space in the frontal area of the rat’s head. This area is densely vascularized, and the sensor implanted here does not move during behavioral activation, providing artifact-free electrochemical recording. Both sensors were secured with dental acrylic to three stainless steel screws threaded into the skull. During the same surgery, rats were implanted with a chronic jugular catheter, which ran subcutaneously to the head mount. Rats for the second experiment underwent a similar surgery of iv catheter implantation but the electrochemical sensor was implanted in the SNr [anteroposterior − 5.2, mediolateral 2.0–2.2 mm, and dorsoventral − 7.6 mm from the skull surface, according to coordinates of the rat brain atlas^19^. Rats were allowed a minimum of 5 days of post-operative recovery and at least 3 daily habituation sessions (~ 6 h each) to the recording environment. Jugular catheters were flushed daily with 0.2 ml heparinized saline to maintain patency.

### Electrochemical detection of oxygen

For in vivo oxygen detection we used Pinnacle oxygen sensors coupled with high-speed amperometry. The principles of electrochemical oxygen detection, construction of oxygen sensors, and their calibration were described in detail elsewhere^11,12^. Oxygen sensors were calibrated at 37 °C by the manufacturer (Pinnacle Technology) according to a standard protocol described elsewhere^20^; concentration curves were quantitatively analyzed in-house. During calibration, the sensors produced linear current changes with increases in oxygen concentrations within a range of physiological brain oxygen concentrations (0–20 μM). Substrate sensitivity of oxygen sensors determined as a mean of three 5 μM tests varied from 0.63 to 1.08 nA/1 μM. Oxygen sensors were also tested by the manufacturer for their selectivity toward electroactive substances such as dopamine (0.4 μM) and ascorbate (250 μM), none of which had significant effects on reduction current.

### Experimental procedures

All rats before surgeries were habituated to the environment of future recordings (at least 3 sessions, 6 h each). At the onset of each recording session, rats were briefly anesthetized (< 2 min) with isoflurane and electrochemical sensors were connected to the potentiostat via an electrically shielded flexible cable and a multi-channel electrical swivel. A catheter extension mounted on the cable was used to allow for stress- and cue-free drug delivery from outside the cage. Testing began 90–120 min after the electrochemical sensors were connected to the recording instrument, allowing for baseline currents to stabilize.

For both experiments, we aimed to maintain similar experimental protocols, but in some sessions deviated from this protocol due to technical problems with recording quality from one of two sensors or catheter patency. Typically, during the first recording session (6–8 h), rats were exposed to three arousing stimuli: a short (0.5-s) auditory signal (75 dB), 3-min tail-pinch, and 3-min social interaction with a novel male conspecific. For the tail-pinch trial, a wooden clothespin was manually attached to the base of the tail and removed 3 min later. For social interaction, a novel male rat was introduced into the cage, where the rat was being recorded, and removed 3 min thereafter. Rats were tested with all stimuli twice per session, with each stimulus presented in random order and separated by at least 20 min (for sound) and 60 min (other stimuli). Physiological stimuli were presented when the rats were in quiet resting state with no overt movements and electrochemical currents were relatively stable. During subsequent sessions, the rat was exposed to one natural stimulus (tail-pinch or social interaction) and iv injections of either cocaine (1 mg/kg in 0.3 ml, delivered for 15 s) or heroin at two doses (0.1 mg/kg and 0.4 mg/kg in 0.15 ml and 0.6 ml delivered for 10 and 30 s, respectively. Cocaine and heroin at a lower dose were delivered with 60-min inter-injection intervals while recording following a larger heroin dose continued for two hours. As shown previously, such inter-injection intervals are sufficient for restoration of both the behavioral, physiological and neurochemical responses to cocaine and heroin at these doses. Similar to natural stimuli, drugs were delivered when the rats were in a quiet resting state. A total duration of recording sessions varied between 7 and 8 h.

The 1.0 mg/kg for cocaine is an optimal dose maintaining rat self-administration^21–23^ and well within the range of human consumption. As shown previously, cocaine at this dose modestly increases NAc oxygen levels^14,16^. 0.1 mg/kg of heroin is an optimal dose maintaining rat self-administration^22,24^; with 0.4 mg/kg corresponding to upper limits of human consumption. Heroin at both doses induces a biphasic oxygen response–a transient decrease followed by an increase^18^. The initial decrease that results from respiratory depression is weak and cannot be evident at 0.1 mg/kg and is greatly enhanced at the 0.4 mg/kg dose. Treatment sessions for drugs were counter-balanced; if the animal underwent tail-pinch and cocaine trials in one session, the rat was exposed to social interaction and heroin during the subsequent session. At the end of each session, rats were lightly anesthetized (Equithesin, 0.6–0.7 ml by slow, 1-min injection) and disconnected from the potentiostat. Rats were allowed to recover from anesthesia and their jugular catheters were flushed with sterile saline before being returned to the animal colony.

### Histological verification of electrode placements

When experiments were complete, rats were deeply anesthetized with isoflurane, decapitated, and their brains were extracted and stored in 10% formalin solution. Later, the brains were cut on a cryostat and analyzed for verification of the locations of cerebral implants and possible tissue damage at the area of electrochemical recording.

### Data analysis

Electrochemical data were analyzed in a slow (1-min) and rapid (4-s) time resolution. Because each individual sensor differed in substrate sensitivity, currents were first converted into concentrations (μM) based on sensitivity calibrations provided by the manufacturer and then into percent (%) changes in oxygen concentration. Values from one-minute prior to stimulus presentation and drug injections were averaged and set as the 100% baseline for slow and rapid time-course analyses. One-way repeated measure ANOVA (followed by Fisher LSD post-hoc tests) was used to evaluate statistical significance of stimulus and drug-induced oxygen responses. To assess the relationships between oxygen changes in the NAc and SC space, we used time-dependent correlation analyses to how oxygen changes in one location relate to oxygen changes in another location. For text clarity, quantitative results of most statistical evaluations (F values of one-way repeated measure ANOVAs) and numbers of averaged responses are shown in the Supplemental Materials.

## Results

In this study, we describe the results of two electrochemical experiments conducted in awake, freely moving rats. In the first experiment (6 rats), we simultaneously monitor oxygen levels in the NAc and SC space. Rats for this experiment were chronically implanted with two oxygen sensors and an iv catheter and then subsequently exposed, during several repeated sessions, to three different arousing stimuli and iv injections of cocaine and heroin. To examine to which extent oxygen responses found in the NAc are structure-specific or reflect a generalized brain phenomenon, in the second experiment rats (n = 4) were implanted with an iv catheter and one oxygen sensor in the SNr. These animals were exposed to a similar experimental protocol as in the first experiment.

### Oxygen responses in the NAc and SC space induced by arousing stimuli

**Figure 1:**
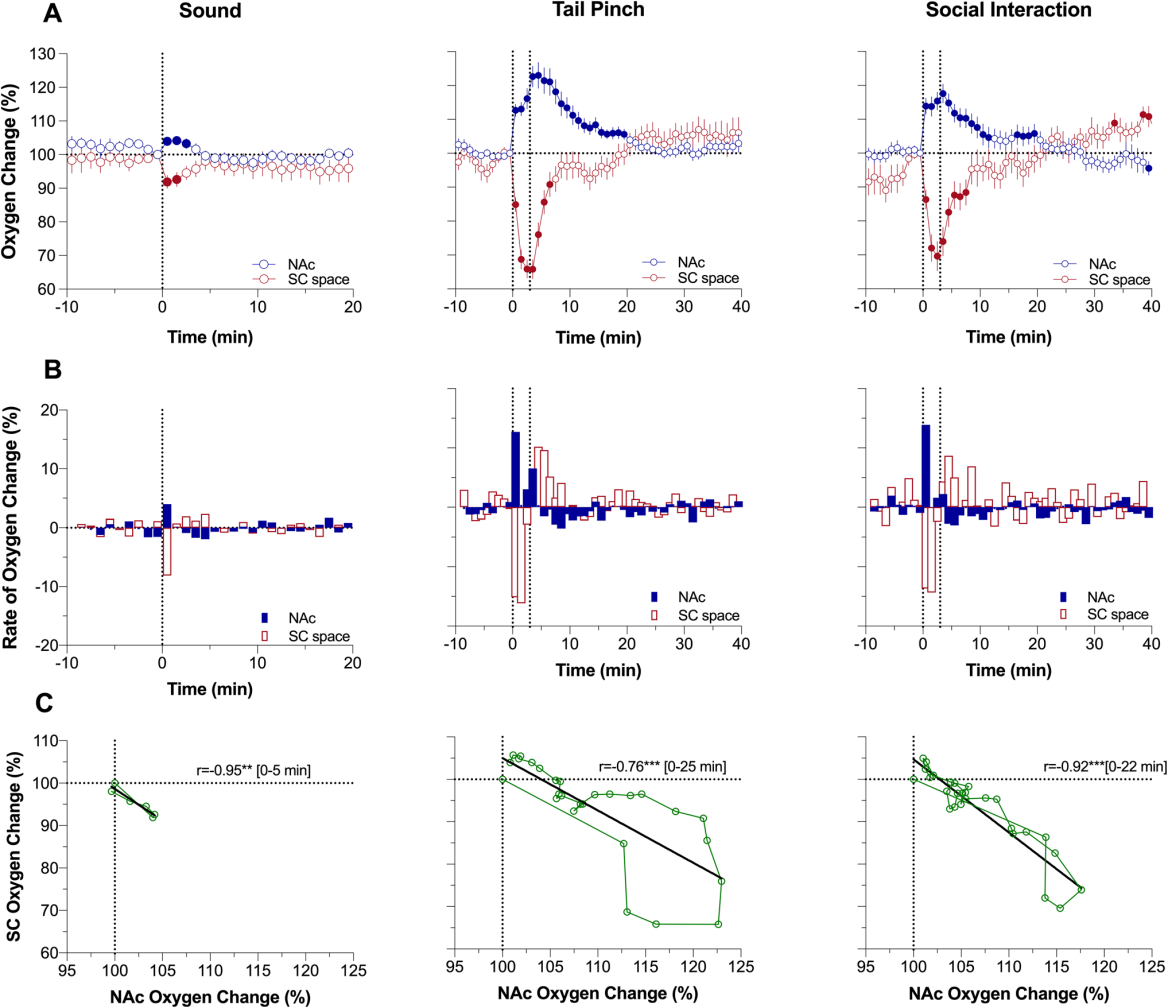
Relationship between changes in oxygen levels in the nucleus accumbens (NAc) and subcutaneous (SC) space induced by auditory stimulus (Sound), Tail-pinch and Social Interaction in awake, freely moving rats. Slow time-course analysis. (**A**) Mean (± SEM) changes in oxygen levels relative to baseline (100%) calculated with 1-min time bins. Vertical hatched lines show onset and offset of stimulus presentation. Filled symbols mark values significantly different from pre-stimulus baseline (*P* < 0.05). (**B**) Rate of oxygen changes. (**C**) Correlative relationships between oxygen changes in two recording locations. r = coefficient of correlation calculated for the time interval of significant increase in NAc oxygen levels. Asterisks show significance of coefficients of correlation (** and ***, *P* < 0.01 and 0.001, respectively). Data were obtained in 6 rats; the numbers of averaged responses in each group are 9–16 (see Table for ANOVA values and n in each group in Supplementary materials).

To represent the entire effects of arousing stimuli on oxygen changes in the NAc and SC space, we first determined percent changes in both parameters averaged with 1-min time-resolution (Fig. 1A). Following this analysis, we found that each stimulus induces significant increases in NAc oxygen levels (F_9,189_ = 2.47, *P* < 0.01; F_13,533_ = 19.60, *P* < 0.001; and F_8,328_ = 14.62, *P* < 0.001 for sound, tail-pinch and social interaction, respectively). All subsequent quantitative results of statistical evaluations of oxygen responses are shown in Supplemental Materials. The effect was weakest for sound (~ 4% peak increase above baseline for 4 min) and larger and more prolonged for tail-pinch (+ 23% at peak for ~ 26 min) and social interaction (~ 18% at peak for ~ 28 min). In each case, the largest rate of brain oxygen increase occurred during the first minute following the stimulus onset (Fig. 1B). An auditory stimulus induced a monophasic increase, but an additional, weaker increase was seen after the offset of both tail-pinch and social interaction. Oxygen changes in the SC space showed opposite dynamics, with larger and more prolonged monophasic decreases (Fig. 1A). Similar to the NAc, the largest rate of changes for all stimuli in the SC space occurred during the first and second minutes following the stimulus onset (Fig. 1B). Changes in NAc and SC oxygen levels for the time of NAc oxygen increase showed strong opposite correlation (Fig. 1C; r = − 0.95, 0.76 and 0.92 four sound, tail-pinch and social interaction, respectively; *P* < 0.001 for all stimuli).

**Figure 2:**
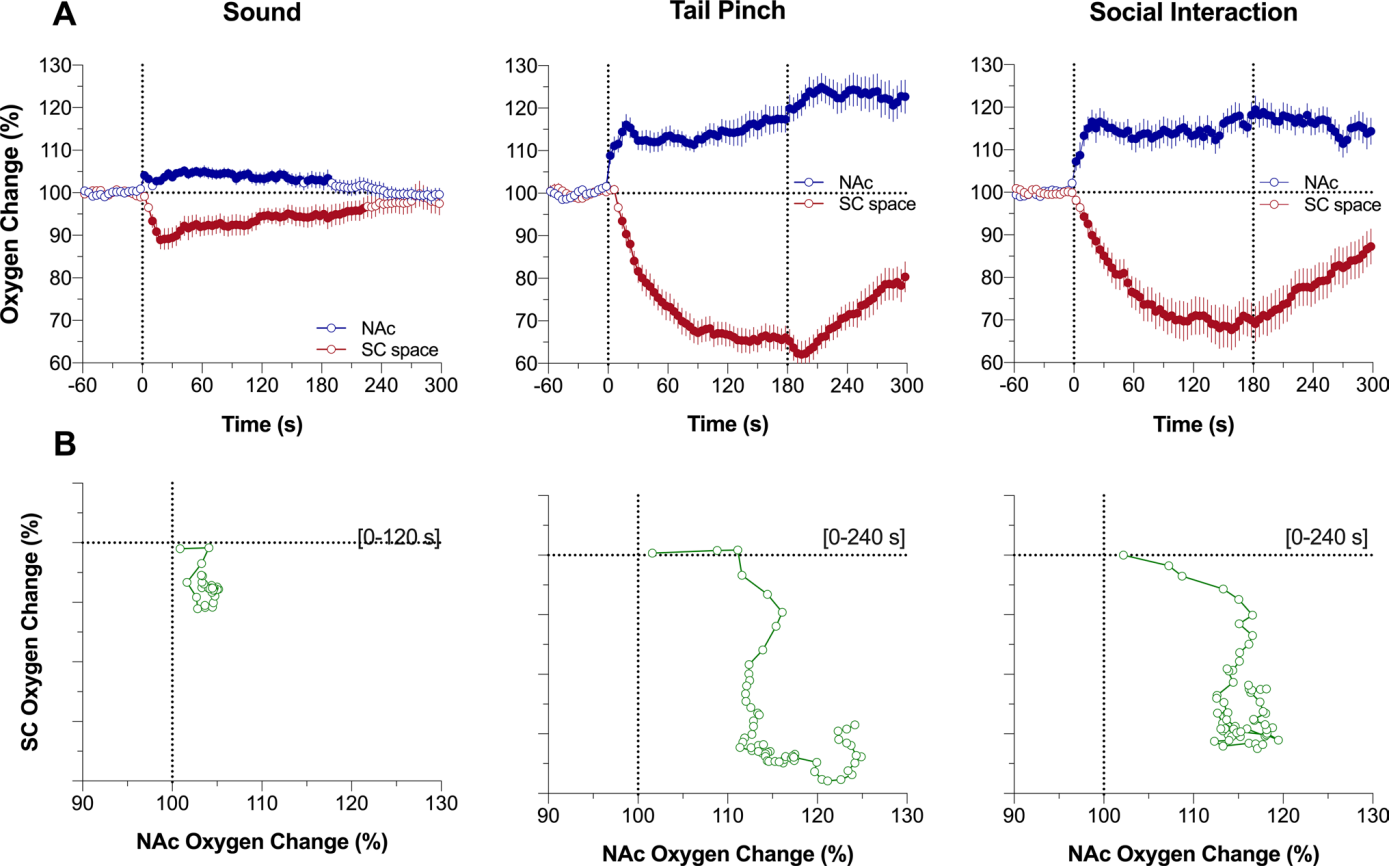
Rapid time-course analysis of relationships between changes in oxygen levels in the nucleus accumbens (NAc) and subcutaneous (SC) space induced by arousing stimuli in awake, freely moving rats. (**A**) Mean (± SEM) changes in oxygen levels relative to baseline (100%) calculated with 4-s time bins. Vertical hatched lines show onset and offset of stimulus presentation. Filled symbols mark values significantly different from pre-stimulus baseline (*P* < 0.05). (**B**) Correlative relationships between oxygen changes in two recording locations shown for the time interval between the stimulus onset and maximal oxygen increase (duration of this interval is shown in brackets). Data were obtained in 6 rats; the numbers of averaged responses and F values of one-way repeated measure ANOVAs are shown in Supplementary materials.

For precise assessments of differences in oxygen dynamics in both recording locations, we used rapid time-course analyses, when the initial period following stimulus presentation (5 min) was analyzed with 4-s time bins (Fig. 2A). In this case, we found that NAc oxygen increase is exceptionally rapid, showing significance from the first time bin (0–4 s) following the onset of each stimulus. In contrast, changes in SC oxygen levels occurred more slowly, with the first significant values at the 8–12 s (sound and social interaction) or 12–16 s (tail-pinch) intervals following the stimulus onset. To better represent the location-specific relationships between oxygen responses, we used time-dependent correlative analysis, which shows how changes in one parameter depend on changes in another parameter (Fig. 2B). In this case, we found that changes in SC oxygen levels occurred with consistent delay (~ 6-s for sound and ~ 10 s for tail-pinch and social interaction) following more rapid changes in brain oxygen.

### Oxygen responses in the NAc and SC space induced by cocaine

**Figure 3:**
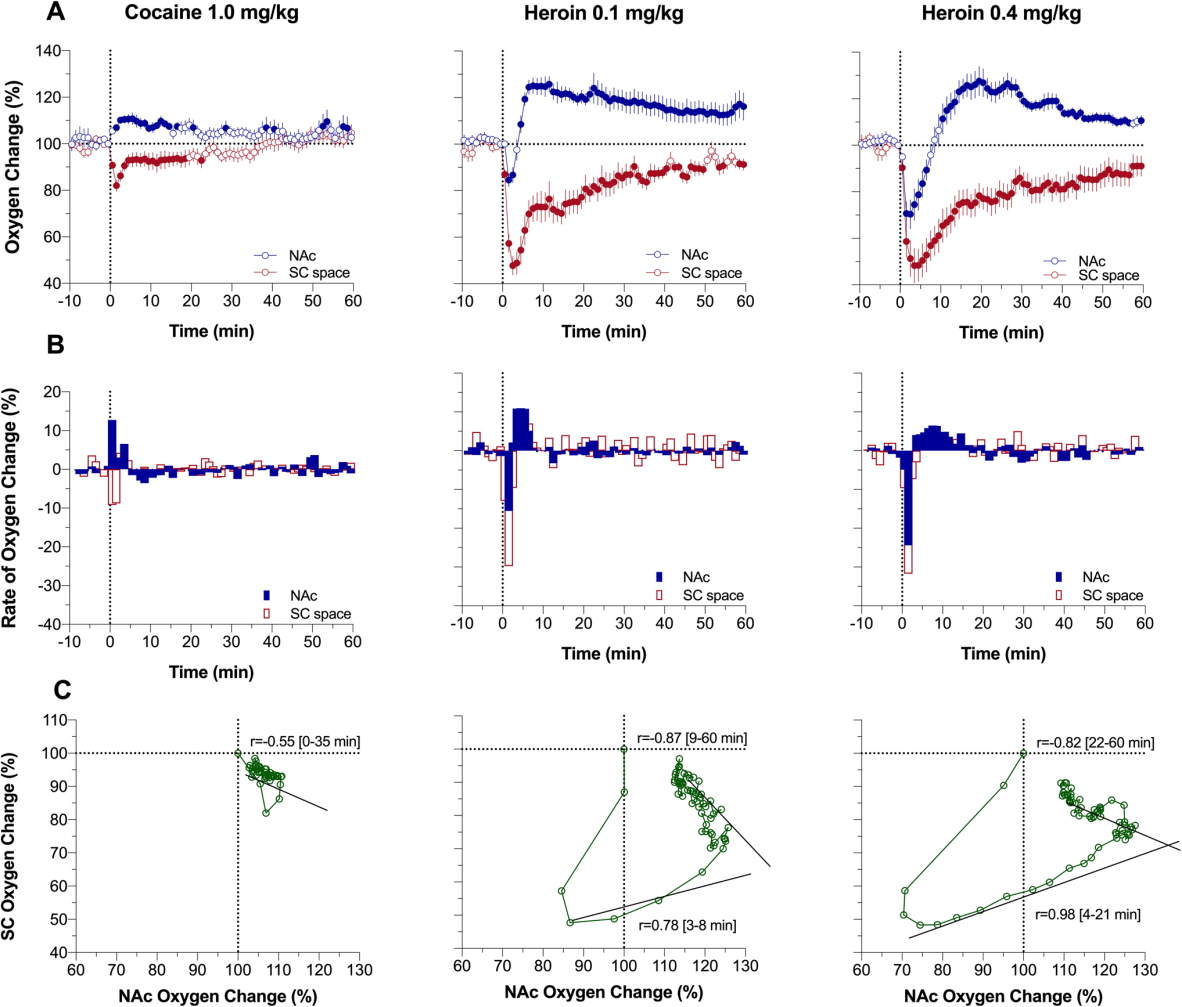
Relationships between changes in oxygen levels in the nucleus accumbens (NAc) and subcutaneous (SC) space induced by intravenous cocaine and heroin in awake, freely moving rats. Slow time-course analysis. (**A**) Mean (± SEM) changes in oxygen levels relative to baseline (100%) calculated with 1-min time bins. Vertical hatched lines show onset and offset of stimulus presentation. Filled symbols mark values significantly different from pre-stimulus baseline (*P* < 0.05). (**B**) Rate of oxygen changes. (**C**) Correlative relationships between oxygen changes in two recording locations shown for time intervals of brain oxygen increases. Coefficients of correlation for heroin (r) are shown for two time intervals: brain oxygen increase from its lowest point to peak and subsequent return to baseline (timing of these intervals are shown in brackets). Asterisks show significance of coefficients of correlation (** and ***, *P* < 0.01 and 0.001, respectively). Data were obtained in 6 rats; the numbers of averaged responses are 11–14 (see Table in Supplementary materials for ANOVA values and n in each group).

As shown in Fig. 3A, iv cocaine at a 1 mg/kg dose significantly increased brain oxygen levels (~ 11% above baseline at peak) and decreased SC oxygen levels (82% of baseline at nadir). This pattern was virtually identical to those induced by sensory stimuli, but changes were weaker but more prolonged than those elicited by tail-pinch and social interaction. Similar to arousing stimuli, the maximal rate of increase was at the first minute following the injection start (Fig. 3B) and changes in NAc and SC oxygen showed significant negative correlation (Fig. 3C; r = − 0.55, *P* < 0.01).

**Figure 4:**
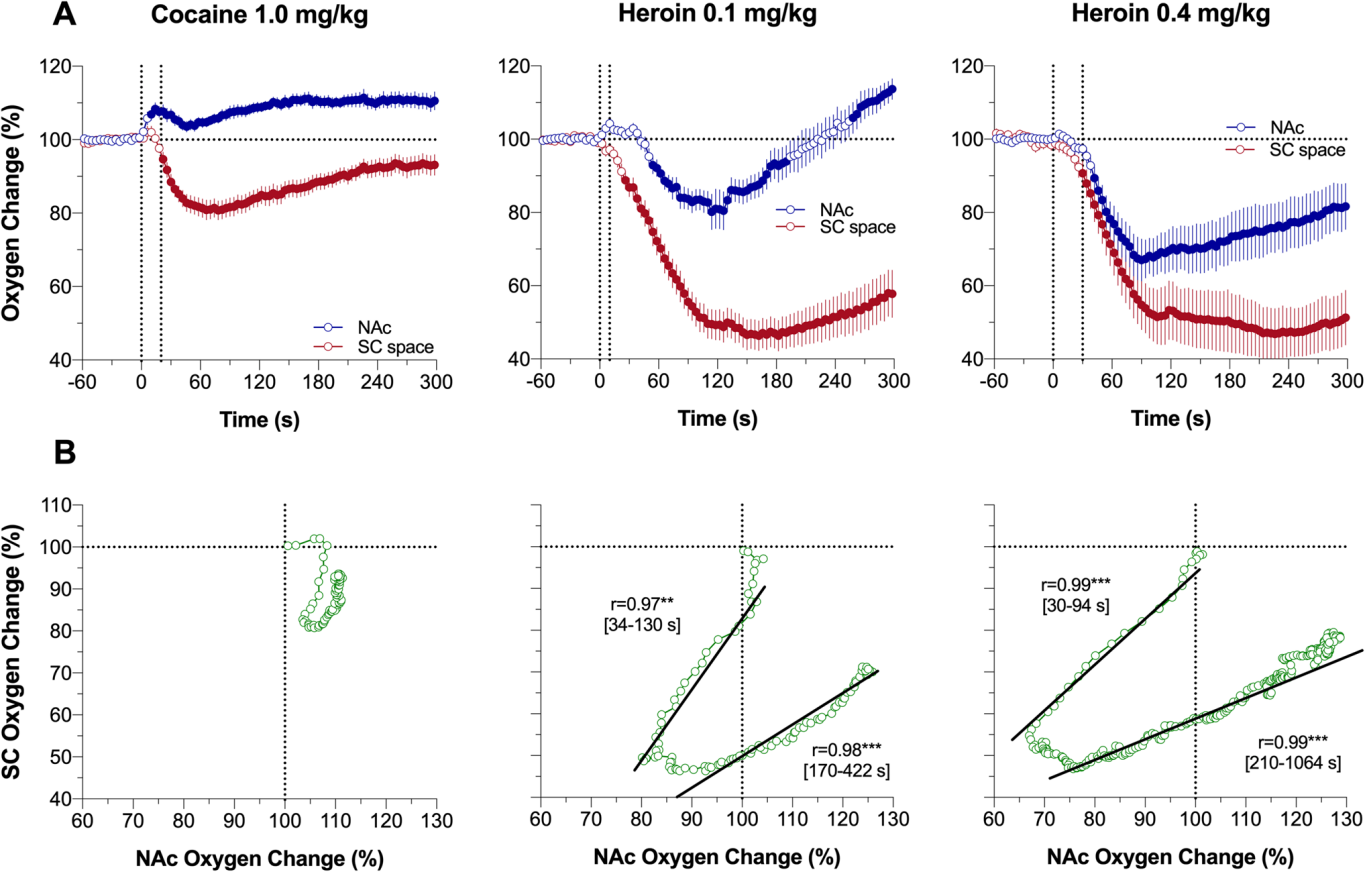
Rapid time-course analysis of relationships between changes in oxygen levels in the nucleus accumbens (NAc) and subcutaneous (SC) space induced by intravenous cocaine and heroin in awake, freely moving rats. (**A**) Mean (± SEM) changes in oxygen levels relative to baseline (100%) calculated with 4-s time bins. Vertical hatched lines show onset and offset of stimulus presentation. Filled symbols mark values significantly different from pre-stimulus baseline (*P* < 0.05). (**B**) Correlative relationships between oxygen changes in two recording locations shown separately for oxygen decreases and subsequent oxygen increases (timing of these intervals is shown in brackets). Asterisks show significance of coefficients of correlation (***, *P* < 0.001 and 0.001, respectively).

The cocaine-induced brain oxygen response was monophasic at the slow time-resolution analysis, but rapid time-course analysis revealed that this effect is in fact biphasic with an initial transient increase during the injection followed by a more prolonged but decaying increase thereafter (Fig. 4A). The decrease in SC oxygen was stronger in magnitude, reaching its nadir later than the brain oxygen peak but remained monophasic within the entire analysis interval. As revealed by time-dependent correlative analysis, the increase in brain oxygen clearly preceded a later developed decrease in SC oxygen—the pattern found with all arousing stimuli tested. However, no correlation was found for rapid cocaine-induced changes in NAc and SC oxygen levels (Fig. 4B).

### Oxygen responses in the NAc and SC space induced by heroin

Consistent with our previous studies^18^, heroin induced a biphasic change in NAc oxygen levels, with a rapid but transient decrease followed by a prolonged increase well above the initial baseline (Fig. 3A). This effect was dose-dependent, and the initial oxygen decrease was clearly larger and more prolonged at the 0.4 mg/kg dose than the 0.1 mg/kg dose. At both doses, heroin induced robust monophasic oxygen decrease in SC space. This change was clearly larger (~ 50% of baseline) and more prolonged than changes seen with arousing stimuli. SC changes were also more prolonged and stronger than brain oxygen decreases (~ 85% of baseline); these levels did not fully return to baseline at the end of a 1-h analysis interval. From analysis of the rate of oxygen change, we see that the effects of heroin are biphasic, showing a rapid transient drop followed by a more prolonged increase to and above the pre-injection baseline (Fig. 3B). As shown by correlative analysis (Fig. 3C), the relationship between changes in NAc and SC oxygen levels differed from those seen with physiological stimuli and cocaine. Initially, only SC oxygen levels decreased when NAc levels remained stable before both showed parallel changes (positive correlation r = 0.78 and 0.98 for 0.1 and 0.4 mg/kg; *P* < 0.01). Finally, the correlation was inverted (r = − 0.87 and − 0.82, *P* < 0.001) when NAc oxygen levels decreased and SC oxygen increased toward the pre-injection baselines.

More detailed representation of oxygen dynamics was obtained by rapid time-course analysis (Fig. 4A). In this case, the location-dependent differences were most evident, with a rapid drop in SC oxygen followed by a weaker and more delayed drop in brain oxygen. While the decrease in SC oxygen after 0.1 mg/kg heroin dose became significant at the 7th time point (26 s), decrease in brain oxygen became significant only at the 14th time-point, which corresponds to 50 s following the injection start. Despite this delay, further decreases in oxygen dynamics in these two locations were strongly correlative (Fig. 4B; r = 0.97 and 0.98 for descending and ascending parts of the curve; both *P* < 0.001). Strong, but less pronounced differences were seen with heroin at 0.4 mg/kg dose the effect became significant at the 7th time point for the SC space and at the 11th time point for the NAc. Similar to a smaller dose, further changes were highly correlative for both descending and ascending part of the curves (Fig. 4B).

### Common features and structural differences in physiological and drug-induced brain oxygen responses: SNr recordings

Both arousing stimuli and drugs have both global and structure-specific effects on neuronal activity and brain metabolism. Therefore, it can be reasonably assumed that this is true for changes in brain oxygen that could depend on structure-specific changes in neuronal activity and metabolism. To assess to which extent oxygen responses in the NAc are specific for this structure or typical to the brain as a whole, we examined how the same arousing and pharmacological stimuli affect oxygen responses in the SNr. In contrast to accumbal neurons, most of which are silent or have slow, sporadic impulse activity in quiet resting state and show phasic excitations by arousing stimuli^25–28^, SNr neurons are autoactive with high levels of basal activity and transient inhibitions/excitations elicited by sensory stimuli^29–32^.

**Figure 5:**
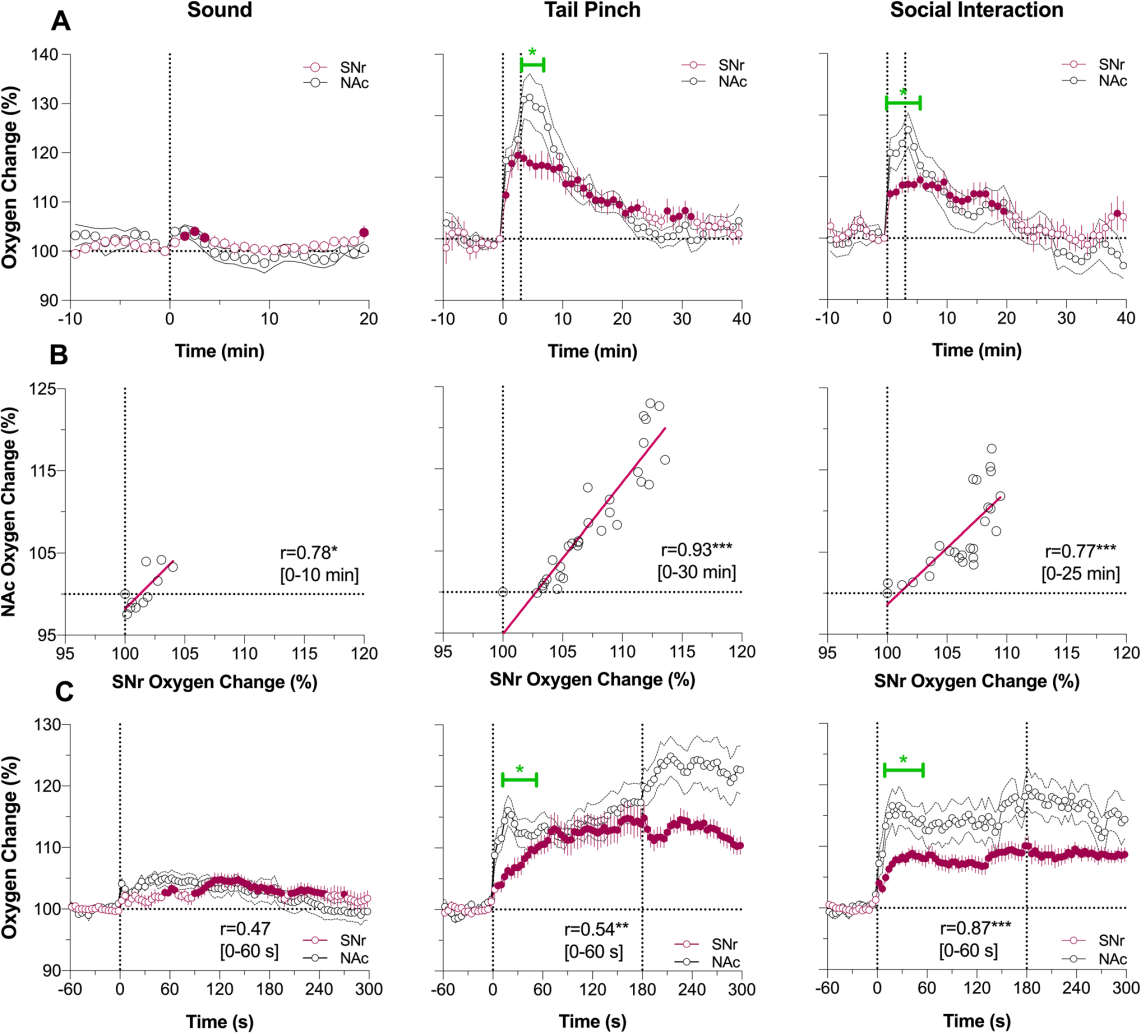
Changes in oxygen levels in substantia nigra, pars reticulata (SNr) induced by auditory stimulus (Sound), Tail-pinch and Social Interaction in awake, freely moving rats. Comparison with nucleus accumbens (NAc) responses. (**A**) Mean (± SEM) changes in oxygen levels relative to baseline (100%) calculated with 1-min time bins. Vertical hatched lines show onset and offset of stimulus presentation. Filled symbols mark values significantly different from pre-stimulus baseline (*P* < 0.05). (**B**) Correlative relationships between oxygen changes in two recording locations. r = coefficient of correlation calculated for the time interval of significant increase in NAc oxygen levels (values in brackets). (**C**) Mean (± SEM) changes in oxygen levels relative to baseline (100%) calculated with 4-s time bins. SNr data were obtained in 4 rats; 12, 9 and 9 responses were averaged for cocaine and heroin at two doses, respectively) (see Supplementary materials for ANOVA values and n in each group). Asterisks show significance of coefficients of correlation (*, ** and ***, *P* < 0.05, *P* < 0.01 and 0.001, respectively). Green line with asterisk shows significant between-structure differences in oxygen curves for the initial period following stimulus presentation.

As shown in Fig. 5A, SNr oxygen responses elicited by arousing stimuli show a pattern identical to that of the NAc, with the smallest transient increase during auditory stimulation with larger and more prolonged increases induced by tail-pinch and social interaction. The increases in SNr oxygen levels were also equally rapid, with maximal rates of change during the first and second minutes of stimulation, peaked at its end, and slowly decayed thereafter. The changes in oxygen levels in both structures tightly correlated (Fig. 5B; r = 0.78, 0.93 and 0.77 for sound, tail-pinch and social interaction, respectively; *P* < 0.05 and *P* < 0.001 for these stimuli), but in the SNr they were more tonic than in the NAc, showing almost two-fold lower peak magnitudes. These structure-specific differences were especially evident when oxygen levels were analyzed with high, 4-s temporal resolution (Fig. 5C). Oxygen increases in the SNr occurred with equally rapid onset latencies (2–6 s) as in the NAc, but they were clearly less rapid with significant between-structure differences at the initial periods of both tail-pinch and social interaction. Despite these differences, rapid oxygen changes elicited by tail-pinch and social interaction in both structures significantly correlated (r = 0.54 and 0.87, respectively; *P* < 0.01).

**Figure 6:**
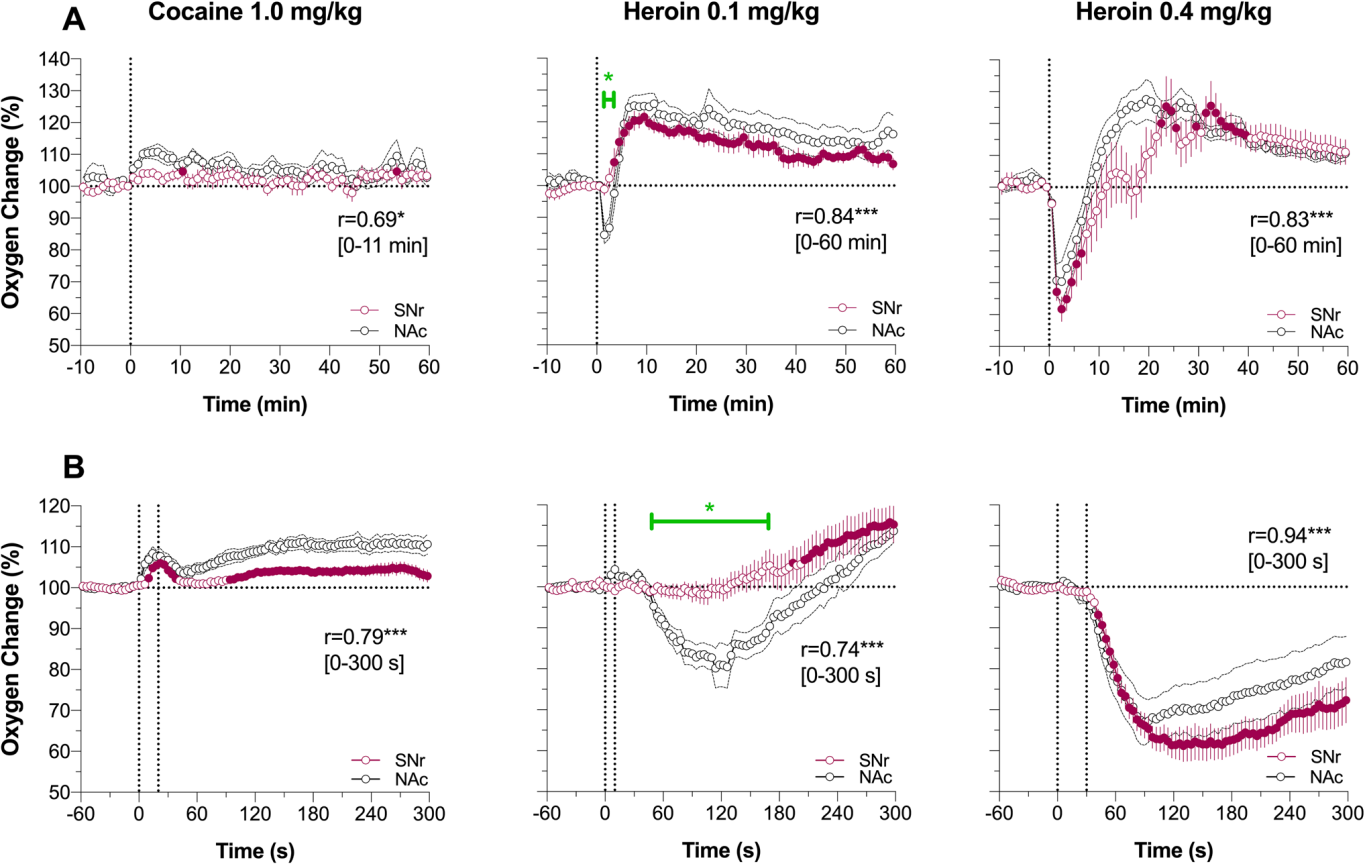
Changes in oxygen levels in substantia nigra pars reticulata (SNr) induced by intravenous cocaine (1.0 mg/kg) and heroin (0.1 and 0.4 mg/kg) in awake, freely moving rats. Comparison with nucleus accumbens data. (**A**) Mean (± SEM) changes in oxygen levels relative to baseline (100%) calculated with 1-min time bins. Vertical hatched lines show onset and offset of stimulus presentation. Filled symbols mark values significantly different from pre-stimulus baseline. r = coefficients of correlations calculated for the time interval to the peak of NAc increase. (**B**) Mean (± SEM) changes in oxygen levels relative to baseline (100%) calculated with 4-s time bins. Green line with asterisk shows significant between-structure differences in oxygen responses. Asterisks show significance of coefficients of correlation (* and ***, *P* < 0.05, and 0.001, respectively). See Supplementary materials for quantitative results of statistical comparisons (group sizes, ANOVAs F values).

Regardless of relatively weak changes in oxygen levels induced by cocaine in the SNr and NAc, they were tightly correlated for both slow (r = 0.69; *P* < 0.05) and rapid (r = 0.79; *P* < 0.001) data analysis (Fig. 6A and B). In both structures the response was bimodal, with a rapid transient increase during the injection followed by a more tonic increase.

Heroin-induced oxygen responses also showed similarity in the SNr and NAc (Fig. 6A and B). While the concentration curves for heroin at a 0.4 mg/kg dose in both structures were superimposable, presenting a very strong correlation (0.91 and 0.94 for 1-min and 4-s analyses; *P* < 0.001), the initial oxygen decrease seen in the NAc was absent in the SNr. Despite this difference in the initial component of oxygen response (1–3 min), both concentration curves significantly correlated for both slow (r = 0.93; *P* < 0.001) and rapid (r = 0.84; *P* < 0.001) time-course analyses.

### Results of verification of sensor locations within the target areas

In our first experiment, we targeted the medial segment of the NAc (“shell”) with our post-experimental evaluation of brain slices confirming that in five of six rats in this group, the sensor tips were located within this subdivision (see Figure S1A in Supplemental Materials). However, in one rat, the sensor’s tip was located more laterally (“core”). To examine whether the recording location may affect the pattern of the oxygen response, we analyzed mean oxygen responses, induced by tail-pinch in each of the six rats of this group (see Figure S1B in Supplementary Materials). This analysis revealed that while there was some between-animal variability, there was a similar pattern in oxygen increase for all six animals. Importantly, the rat with a more lateral sensor location demonstrated an oxygen increase well within the variability of this group. Although SNr was our intendent target in the second experiment, in one of four rats, the sensor tip was located more dorsally at the upper border of the SNr, closer to *SN pars compacta* and parabrachial nucleus (see Fig. S1C in Supplementary Materials). Similar to that in the NAc sample, tail-pinch-induced oxygen increase showed significant variability, but the pattern of oxygen response in the rat with more dorsal sensor location did not differ from that in other rats of this group (see Fig. S1D).

## Discussion

Consistent with our previous studies^11,12^, we show here that brain oxygen levels assessed in the NAc rapidly but modestly increase following exposure to several arousing stimuli of different nature and salience. The primary novel finding obtained with two-sensor oxygen recording in this study is that these physiological brain oxygen increases are accompanied by stronger and more prolonged oxygen decreases in the SC space. Therefore, the enhanced oxygen entry into brain tissue (functional hyperoxia) is associated with relative hypoxia in the periphery. With second-scale temporal resolution analyses, we also see that brain oxygen increases occur with second-scale onset latencies and consistently earlier than decreases in SC oxygen. These findings implicate two factors: direct neural effects on cerebral capillaries and peripheral vasoconstriction that results in redistribution of arterial blood from the periphery to the brain, in mediating cerebral vasodilation/increased CBF as the final cause of physiological brain oxygen increases. The latter factor explains the long duration of brain oxygen increases and the similar response pattern found in two distantly located brain structures. Therefore, functional hyperoxia appears to be a generalized brain phenomenon, with minor between-structure differences evident at high temporal resolution of recordings at early time intervals following the stimulus onset. Finally, we show that the relationships between central and peripheral oxygen changes induced by cocaine and heroin have certain similarities and important differences than those occurring under physiological conditions.

By using second-scale resolution analyses, we found that brain oxygen increases elicited by all tested arousing stimuli occur with very short onset latencies (2–6 s) and preceding oxygen decreases in the SC space. The rapidity and phasic nature of oxygen increases found in the NAc point at neuronal activation as its triggering factor. This dynamic is consistent with the electrophysiological properties of accumbal neurons, most of which are silent or have slow, sporadic spiking activity quiet wakefulness and are phasically excited by arousing stimuli and during motor activity^25–28^. Therefore, the phasic activation of accumbal neurons with subsequent dilation of cerebral vessels and increased CBF, can explain, via the neuro-vascular coupling mechanism, the rapid rise in NAc oxygen levels and its initial concentration peak. Although SNr neurons are tonically autoactive, showing high discharge rate and transient excitations and inhibitions following sensory stimulation^29–32^, oxygen levels in this structure also increased with equally short onset latencies as in the NAc. The pattern of these SNr oxygen responses was also similar to that of the NAc with both concentration curves tightly correlated, which suggests a common neural trigger for both responses. Therefore, similar to increases in CBF found in different brain structures following exposure to various arousing or stressful stimuli^33,34^, brain oxygen increases appear to be a generalized, arousal-related phenomenon occurring in the brain as a whole. However, consistent with the presumed differences in impulse activity, oxygen responses in the SNr were more tonic with weaker concentration increases.

The term “functional neural activation” often used in modern literature to explain cerebral vascular response is not precise as it includes several different components or manifestations. These include: neuronal activation as a phasic electrophysiological event, sympathetic activation as a centrally mediated multi-component autonomic response, and behavioral activation with changes in locomotor and motor activity. Functional neural activation also includes metabolic neural activation that can be quantified based on oxygen consumption or metabolism-related heat production. While metabolic activity of a whole organism could be accurately quantified by calorimetry based on changes in heat production, it is much more difficult to assess dynamically brain metabolic activity under physiological conditions. In our previous studies, multi-site thermorecording was used as an approach to assess metabolic brain activity and peripheral vascular response based on temperature differences in different recording sites^35^. Specifically, changes in temperature differentials between the brain site and temporal muscle (non-locomotor muscle with no metabolic activity by its own) provide a reliable measure of the brain’s heat production as an index of metabolic activity.

Using this approach, we previously showed that the arousing stimuli used in this study induce increases in brain-muscle differentials indicative of metabolic brain activation. Importantly, the pattern of these increases was similar in different brain structures, suggesting a generalized phenomenon, with minimal between-structure differences evident only with high temporal resolution analyses^35,36^. The time-course of these increases in NAc-muscle differentials was generally similar to that of oxygen responses in this study, but developed with longer onset latencies and were more tonic and shorter in duration. Therefore, it appears that enhanced entry of oxygen into brain tissue precedes increases in metabolic brain activation, thus providing additional oxygen to prevent its possible decreases from a later occurring metabolic consumption. Similarly, rapid “anticipatory” changes preceding slower increases in brain metabolic activity were previously shown for various arousing stimuli with glucose, another essential substance also arriving into brain tissue from the arterial blood^37^. Therefore, the rise in brain levels of oxygen and glucose is not a response to increases in consumption during functional neural activation, but anticipatory change to prevent any possible metabolic deficit.

While neuronal activation elicited by arousing stimuli appears to be the initial trigger for brain oxygen increases, these increases are relatively prolonged and greatly exceed the duration of stimulation, suggesting the contribution of other more tonic mechanisms. Previous thermorecording studies revealed that arousing stimuli decrease skin temperature and skin-muscle differentials indicative of skin vasoconstriction^36^. While this effect is centrally mediated, being a component of sympathetic activation, by inhibiting heat dissipation to the external environment, it provides a major contribution to brain and body temperature increases induced by these stimuli. As shown here, all arousing stimuli tonically decreased SC oxygen levels and skin vasoconstriction appears to be the only cause for these decreases. While the direct neural effects on brain capillaries of a number of vasoactive substances released by neurons and glia (i.e., NO, CO2, adenosine, etc.) are usually viewed as the primary mechanism underlying cerebral vasoconstriction and increased CBF^1–3^, our current findings with SC oxygen measurements underscore the important contribution of peripheral vasoconstriction in mediating these effects, thus explaining the similarity in the general pattern of oxygen responses in different brain structures and relatively long duration of these responses. By constricting skin vessels and decreasing blood flow in the periphery, more arterial blood is entering brain tissue (functional hyperemia), providing more oxygen and glucose necessary for enhanced metabolic activity. Thus, it appears that peripheral vasoconstriction has dual functional significance: as a factor inhibiting heat dissipation and inducing functional hyperthermia and a factor responsible for blood redistribution from the less important for survival peripheral organs to more important for survival brain and heart.

Decreases in SC oxygen levels assessed with a minute time scale showed strong negative correlation with brain oxygen levels. However, rapid time-course analyses revealed that these changes consistently follow brain oxygen increases but are more tonic than changes in brain oxygen. This dynamic is consistent with the mechanisms underlying this effect, pointing at the role of epinephrine and norepinephrine as primary factors mediating peripheral vasoconstriction and thus adaptive redistribution of blood between central and peripheral domains that prepares the organism for “fight or flight.” In support of this mechanism, norepinephrine at very low doses (2–4 μg/kg), by constricting peripheral vessels, phasically increases brain levels of oxygen and glucose^38^.

As shown in this study, iv cocaine at an optimal self-administering dose generally mimicked the pattern of oxygen responses in the NAc and SC space induced by arousing stimuli. However, these responses were smaller in magnitude and more prolonged in duration. While cocaine is a known vasoconstrictor, at this low dose the magnitude of drug-induced peripheral vasoconstriction assessed by decreases in SC oxygen levels was weaker than that induced by salient arousing stimuli. Cocaine and salient arousing stimuli (tail-pinch and social interaction) also show similar changes in EEG and EMG activity^39^, similarly modestly increase brain temperature^40^ and induce comparable locomotor activation^40^. Therefore, iv cocaine passively administered to quietly resting freely moving rats at low, self-administering doses can be viewed as pharmacological arousing stimulus.

Similar to natural arousing stimuli, cocaine-induced brain oxygen increases occurred with exceptionally rapid onset latencies (8–12 s), which is difficult to explain as the direct effect on brain receptive substrates. Time is necessary for the drug to travel from the injection site to brain vessels, cross three-layers of the blood–brain barrier (BBB), passively diffuse to brain receptive substrates and interact with them. As confirmed with cocaine-methiodide, a BBB-impermeable cocaine analog, the initial rapid component of bimodal oxygen response is mediated by cocaine’s action on receptive sites on afferents of peripheral sensory nerves, while subsequent tonic effects are mediated by the direct action of cocaine on brain receptive substrates (see for details and further discussion^41^). As shown in this study, the start of the second, more tonic phase of NAc oxygen increase occurred at ~ 52 s from the injection start, suggesting the appearance of a direct central effect. Interestingly, the onset of heroin-induced NAc oxygen decrease occurred at the same time (~ 52 s), which may reflect the duration of drug travel to centrally located opioid receptors.

In contrast to physiological stimuli and cocaine, heroin induces dose-dependent respiratory depression that can result in coma and even death at high drug doses^42–45^. Therefore, it is not surprising that oxygen levels in both the NAc and SC space initially decreased with a clearly larger and more prolonged decrease in the SC space. In contrast to natural stimuli and cocaine, the decrease began from the SC space and continued, with a certain delay, in the brain showing a strong between-site correlation both for the decreasing and ascending parts of the curves. While decreases in the SC space were strong and monophasic, the effect was weaker in the brain where oxygen levels showed a strong rebound-like oxygen increase. The latter change suggests that the brain engages in some compensatory mechanisms to eliminate the consequences of global hypoxia that results from respiratory depression. Loss of oxygen, accumulation of CO2 and the release of other vasoactive substances are the possible factors determining the post-hypoxic rebound-like cerebral vasodilation and subsequent brain oxygen increase. Therefore, the biphasic brain oxygen response induced by heroin reflects interaction between two independent and opposing mechanisms. Oxygen levels tend to decrease due to rapidly developed respiratory depression and subsequent drop in blood oxygen levels but tend to increase these levels by products of metabolism and previous hypoxia. While the latter factors could play a role in post-hypoxic brain oxygen increase, skin vasoconstriction induced by this drug^46^ is another contributor. Interestingly, heroin at a lower dose (0.05 mg/kg), which is still in the reinforcing range, induces only a weak, monophasic and more tonic increases in NAc oxygen^16^ that could be related to drug-induced peripheral vasoconstriction. Therefore, brain hypoxia resulting from respiratory depression is an effect typical only for relatively large heroin doses.

### Concluding Remarks and Functional Implications

Since both oxygen and glucose arrive to the brain from arterial blood, the primary focus of numerous studies was on different aspects of brain blood supply (tone of cerebral vessels and CBF) associated with functional neural activation. Although these studies reveal a basic association of increased CBF with neural activation, most technologies used in these studies are complex, indirect, have low temporal resolution, and cannot be used in freely moving animals, especially rats. Development of electrochemical techniques that provide high substrate sensitivity, selectivity, and second-scale temporal resolution make it possible to assess real-time fluctuations of oxygen and glucose in selective brain areas and body locations in awake, freely moving rats^12^. While the assessments of CBF and the tone of cerebral vessels are important for understanding of how oxygen and glucose arrive to brain capillaries, real-time dynamics of these substances in the brain’s extracellular space and its relationships with fluctuations of these substances in peripheral tissues remained unclear.

The two-sensor recording paradigm allowed us to evaluate the relationships between oxygen changes in the brain and periphery that occur under physiological conditions and following exposure to two widely used drugs of abuse. While most studies assessing CBF are focused on unique features of individual brain structures and between-structure differences, simultaneous high-resolution measurements from the brain and SC space provided several new findings to understand basic mechanisms underlying oxygen fluctuations occurring in the brain. Two findings are especially important. First, oxygen increases in brain tissue induced by arousing stimuli (functional or arousal-related hyperoxia) are tightly correlated with oxygen decreases (i.e., relative hypoxia) in the periphery. This suggests the ability of the brain under conditions of potential danger to redistribute blood supply from less important for survival periphery to the brain. Second, we clarified the role of peripheral vasoconstriction as a cause for SC oxygen decreases and a significant contributor to brain oxygen responses that determines their basic similarity in different brain structures. Peripheral vasoconstriction is also a primary contributor to arousal-related brain hyperthermia. The increases in brain temperature elicited by arousing stimuli also show similar time-course in different brain structure and also oppositely correlate with SC oxygen decreases. While neuronal activation is the initial trigger for rapid, structure-specific brain oxygen increases and subsequent changes in physiological parameters, these initial brain oxygen increases are greatly amplified by enhanced inflow of arterial blood due to peripheral vasoconstriction. This dual mechanism also determines intra-brain entry of glucose, which rapidly increases by arousing stimuli and shows between-structure response similarity and basic correlation with brain oxygen levels^37,47^.

While these basic conclusions are supported by experimental evidence, some could be viewed as assumptions because of the lack of available data on patterns of structure-specific changes in neuronal activity and responsiveness to arousing stimuli. In order to fully understand the mechanisms determining fluctuations of brain oxygen levels under physiological conditions and different pathological states, further studies using different techniques and new approaches will be necessary to verify and clarify these findings.

## Supplementary Information

Supplementary Information

## Data Availability

Raw data and the results of their primary analyses are available on request.
