# Supplementary material for: Relationships between oxygen changes in the brain and periphery following physiological activation and the actions of heroin and cocaine
Source: Sci Rep. 2021 Mar 18;11:6355. doi: 10.1038/s41598-021-85798-y (PMC7973713; doi:10.1038/s41598-021-85798-y)
Supplement: Supplementary file 1 — Supplementary Information [file 41598_2021_85798_MOESM1_ESM.pdf]

Supplementary Information

Relationships between oxygen changes in the brain and periphery following physiological activation and the actions of heroin and cocaine

Shruthi Thomas, Carlos M. Curay, Eugene A. Kiyatkin*

Behavioral Neuroscience Branch, National Institute on Drug Abuse – Intramural Research Program, National Institutes of Health, DHHS, Baltimore, MD 21224, USA

Quantitative results of statistical analyses of oxygen responses in three recording locations analyzed with slow (1-min) time resolution

Stimulus	NAc		SC space		SNr	
	n	F value	n	F value	n	F value
Sound	10	$F_{9,189}$=2.47***	14	$F_{13,273}$=0.70	13	$F_{12,252}$=2.11**
Tail-pinch	14	$F_{13,533}$=19.60***	16	$F_{15,615}$=15.10***	13	$F_{13,533}$=11.68**
Social Interaction	9	$F_{8,328}$=14.62***	11	$F_{10,410}$=11.60***	14	$F_{13,533}$=5.918***
Cocaine 1 mg/kg	16	$F_{9,549}$=1.24	16	$F_{15,915}$=4.63***	12	$F_{11,671}$= 0.84
Heroin 0.1 mg/kg	13	$F_{12,732}$=7.47***	11	$F_{10,610}$=13.78***	9	$F_{8,488}$= 6.93***
Heroin 0.4 mg/kg	14	$F_{13,793}$=15.80***	12	$F_{11,617}$=16.38***	9	$F_{8,488}$= 6.73***

n=number of averaged tests; F=ANOVA F value with degrees of freedoms; *, **, *** = the level of significant change with p<0.05, 0.01 and 0.001, respectively.

Quantitative results of statistical analyses of oxygen responses in three recording locations analyzed with rapid (4-s) time resolution

Stimulus	NAc		SC space		SNr	
	n	F value	n	F value	n	F value
Sound	10	$F_{9,684}$=4.94***	14	$F_{13,988}$=4.20***	13	$F_{12,912}$= 1.61**
Tail-pinch	14	$F_{13,988}$=12.60***	16	$F_{15,1140}$=26.96***	13	$F_{13,988}$=6.36***
Social Interaction	9	$F_{8,608}$=3.70***	11	$F_{10,760}$=17.02***	14	$F_{13,988}$=4.31***
Cocaine 1 mg/kg	16	$F_{9,684}$=4.99****	16	$F_{15,1140}$=15.16***	12	$F_{11,836}$=3.79***
Heroin 0.1 mg/kg	13	$F_{12,912}$=21.66***	11	$F_{10,760}$=38.69***	9	$F_{8,608}$= 7.90***
Heroin 0.4 mg/kg	14	$F_{13,988}$=10.73***	12	$F_{11,836}$=24.79***	9	$F_{8,608}$=30.02***

n=number of averaged tests; F=ANOVA F value with degrees of freedoms; *, **, *** = the level of significant change with p<0.05, 0.01 and 0.001, respectively.

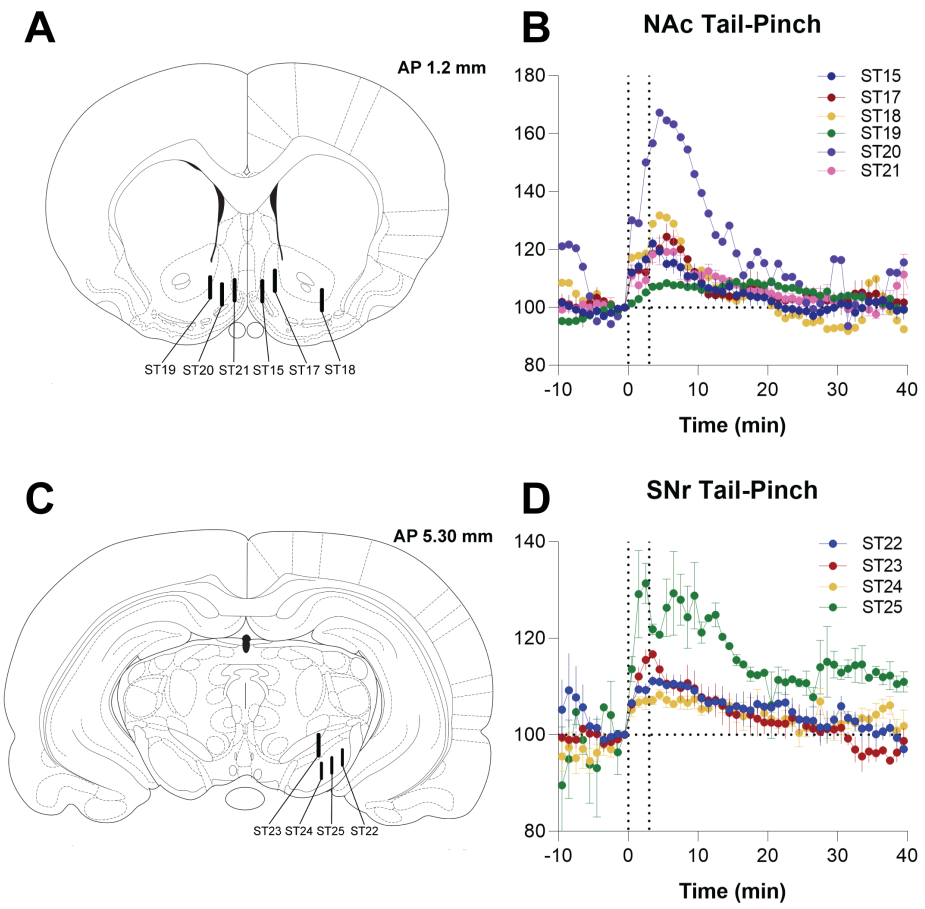

Figure S1. Results of histological verification of sensor locations in all rats in the NAc and SNr groups and individual differences in oxygen responses induced by tail-pinch in all tested rats. In all rats of the first experiment (n=6), sensor tips were located within the NAc and in all rats, tail-pinch induced increases in oxygen levels. The right panel shows reconstruction of sensor locations for each rat in both experiments (A=NAc, C=SNr). The left panel shows mean changes in oxygen levels induced by tail-pinch for each rat in both experiments (B=NAc, D=SNr). While the sensor tip of rat ST18 was located more laterally than other rats of the NAc group, tail-pinch induced changes in oxygen levels were well within group variability and did not differ significantly from other rats of this group. We see a large oxygen increase in rat ST20, for which the sensor top was located in the NAc shell. The sensor tip of rat ST23 was located slightly above the SNr, closer to SNc, zona inserta, and parabrachial nucleus. Despite a presumed off-target location, changes in oxygen induced by tail-pinch of this rat did not differ from changes found in other rats of this group. The reasons for a stronger oxygen change in rat ST25, for which the sensor tip was located within the SNr, remain unclear.
